# AI for precision epilepsy: ASM response prediction needs real-world validation

**DOI:** 10.1097/MS9.0000000000004424

**Published:** 2025-11-25

**Authors:** Areeba Naveed, Hamza Sajid, Noor Un Nisa Irshad, Sakan Binte Imran

**Affiliations:** a Department of Medicine, Allama Iqbal Medical College, Lahore, Pakistan; b Department of Medicine, Jinnah Sindh Medical University, Karachi, Pakistan; c Department of Medicine, Sir Salimullah Medical College, Dhaka, Bangladesh

*To the Editor,*

Dravet syndrome (DS) is a serious developmental and epileptic encephalopathy that begins in infants who were born healthy. It represents a subset of severe epilepsies with recurring, difficult-to-treat seizures, and severe developmental delays. The most frightening part of DS is the high risk of sudden unexpected deaths in epilepsy^[1]^. This disorder is primarily caused by a mutation in the sodium channel gene SCN1A. Seizures usually occur between 2 and 15 months of age and are often induced by fever. Seizures in DS are frequently prolonged and are not well managed by current medications.

This condition is rare, affecting 1 in every 15 000 to 40 000 babies. Unfortunately, about 20–25% of patients do not survive into adulthood. Combined with deaths due to status epilepticus and other complications, the cumulative mortality rate emphasizes the imperative, unmet demand for successful therapeutic approaches. Treatment response heterogeneity is extreme, resulting in families reliving a long, traumatic series of trials of antiseizure medication (ASM) with limited efficacy and often debilitating side effects.

Under these high-stakes clinical conditions, artificial intelligence (AI) offers a beacon of hope. Evidential studies have shown that machine learning models can predict a person’s ASM response, opening the way for a precision medicine approach that could bring closure to the therapeutic odyssey for these children^[2]^. The central concept is that machine learning models can analyze complex, multidimensional patient data such as genetic subtypes, neuroimaging findings, and past responses to therapy. This is the epitome of precision medicine, and it promises to enable clinicians to bypass years of unproductive and detrimental polypharmacy. Early evidence-based studies are promising, e.g., a machine learning model retrospectively predicted medication response with 87.5% accuracy for focal epilepsy^[3]^. Applying this approach to DS could mitigate the ever-looming threat of mortality.

However, this promise is moderated by a critical peril. The validation of these AI models in current practice is overwhelmingly reliant on small, retrospective, and carefully selected datasets^[4]^. These clean datasets are scrubbed of the complexities of real-world clinical practice: polypharmacy, comorbidities, variable patient adherence, and missing data entries. This creates a perilous translational gap between algorithmic performance in a controlled research environment and reliable efficacy at the patient’s bedside^[5]^. Most importantly, there is a stark lack of prospective evidence to show that AI-aided ASM selection benefits actual DS patients^[6]^. High accuracy in a model on past data is a scientific wonder, not an established therapy.

The consequences of this validation gap are serious. Firstly, it poses irreversible and direct clinical danger. An ineffective and poorly calibrated model implemented in practice can lead to failed treatment, prolonged risk of seizures, and increased risk of catastrophic outcomes such as status epilepticus or SUDEP. Secondly, early release of untested tools risks a disastrous breakdown in trust between the clinicians who will have to work with them and the families who hold their hopes in them^[7]^. This loss of confidence would bring progress in the entire field of AI in neurology to a standstill for years. Finally, it is a substantial misallocation of limited resources, taking attention, skill, and dollars away from more direct and established patient-benefit studies.

To bridge this gap, the research community must shift its focus from a narrow pursuit of algorithmic novelty to a dedicated mandate of rigorous, prospective validation. Immediate priority should be accorded to randomized controlled trials (RCTs) in which patients are prospectively randomized to either AI-driven or conventional-care ASM choice. Since deep drug resistance is the key problem in DS treatment^[8]^ and, on the other hand, overcoming it is an achievable objective. These trials need to intentionally transcend the barrier of surrogate endpoints and instead measure meaningful and patient-important outcomes: sustained seizure freedom rates, reduced emergency room visits, and measurable improvements in quality of life. This article aligns with the TITAN Guidelines on the need for transparency in AI use in healthcare^[9]^.

In conclusion, the present use of AI in DS is an intriguing hypothesis, not a proven therapy. For the benefit of at-risk children and their families, we must move beyond the sole focus of creating smarter algorithms and toward delivering smarter care. The road to algorithmic promise and actual patient relief must be paved with strong, real-world validation.

## Data Availability

Not applicable.
